# Mesothelin-targeted CAR-T cells secreting NKG2D-BiTEs exhibit potent efficacy against triple-negative breast cancer

**DOI:** 10.1186/s40164-025-00621-y

**Published:** 2025-03-03

**Authors:** Muhammad Auwal Saliu, Qi Wang, Mansur Dabai Salisu, Yuanfeng Ren, Pengchao Zhang, Rabiatu Bako Suleiman, Bingbing Cao, Yiqiao Xu, Xudong Liu, Frederic Lluis, Maoxuan Liu, Xiaochun Wan

**Affiliations:** 1 Guangdong Immune Cell Therapy Engineering and Technology Research Center, Center for Protein and Cell-Based Drugs, Institute of Biomedicine and Biotechnology, Shenzhen Institutes of Advanced Technology, Chinese Academy of Sciences, Shenzhen, 518055 China; 2 University of Chinese Academy of Sciences, Beijing, 100049 China; 3 Department of Gastroenterology, Daping Hospital, Army Medical University, Chongqing, China; 4 Hunter Biotechnology, Inc., Hangzhou, 310051 China; 5 The State Key Laboratory for Complex, Severe and Rare Diseases, Peking Union Medical College Hospital, Beijing, 100730 China; 6 Department of Development and Regeneration, Stem Cell Institute, KU Leuven, Leuven, 3000 Belgium

**Keywords:** CAR-T therapy, BiTEs, Triple-negative breast cancer, Mesothelin, NKG2D ligands

## Abstract

Triple-negative breast cancer (TNBC) is an aggressive subtype with poor prognosis and limited treatment options. Chimeric antigen receptor (CAR)-T cell therapy holds promise, but its efficacy is hindered by tumor antigen escape and heterogeneity. To address these challenges, we developed a novel bispecific T cell engagers CAR-T (BiTEs CAR-T) targeting Mesothelin (MSLN) and secreting NKG2D-Bispecific T cell Engagers (BiTEs) to engage NKG2D ligands (NKG2DL). Analysis of TNBC tissues using The Cancer Genome Atlas and tumor microarrays revealed high but weakly correlated expression of MSLN and NKG2DL, making them ideal targets for dual engagement. To reduce immunogenicity and enhance stability, we used a nanobody and the natural receptor NKG2D as antigen-binding domains instead of traditional scFvs in the CAR construct. The secreted BiTEs could promote the cytotoxicity of untransduced T cells against NKG2DL + tumor cells. In vitro, BiTEs CAR-T cells exhibited superior cytotoxicity, T cell activation, and cytokines production against heterogeneous target cells compared to MSLN CAR-T. In vivo, BiTEs CAR-T cells demonstrated potent antitumor activity in zebrafish and murine TNBC models, significantly reducing tumor burden and prolonging survival without detectable toxicity. These findings suggest that BiTE CAR-T cells offer a highly promising therapeutic strategy for TNBC by addressing antigen heterogeneity and immune escape mechanisms, with promising translational potential for clinical application.

**Supplementary Information:**

The online version contains supplementary material available at 10.1186/s40164-025-00621-y.

**To the editor**,

Triple-negative breast cancer (TNBC) is the most malignant subtype of breast cancer with a poor prognosis [1]. Although chimeric antigen receptor (CAR)-T cell therapy has shown promise, its efficacy in TNBC is hampered by tumor antigen escape and heterogeneity [2, 3]. Targeting multiple tumor-associated antigens simultaneously could address these limitations. Bispecific T cell Engagers (BiTEs) redirect bystander T cells to tumor cells and was shown to circumvent antigen escape without detectable toxicity [4]. Therefore, we developed a novel BiTE-secreting CAR-T for TNBC treatment.

Single-target CAR-T cells targeting Mesothelin (MSLN) or NKG2D ligands (NKG2DL) show good safety in clinic, but limited efficacy [5, 6]. Our analysis of TCGA [7], showed high but independent expression of MSLN and NKG2DL in TNBC tissues (Fig. S1A, S1B). Immunohistochemistry validated these findings showing that 71.05% and 55.26% of TNBC samples were positive for MSLN and MICA/B, respectively, with only 15.79% negative for both (Fig. 1A, S1C). MSLN appears to be more specific than NKG2DL, as the latter can be induced by acute infection and chemotherapeutics [8]. Then we designed MSLN-targeted CAR-T cells secreting NKG2D-BiTEs (BiTEs CAR-T). To avoid possible immunogenicity and aggregation risks of scFvs, we used nanobody (VHH) with small, stable structure and low immunogenicity to target MSLN [9, 10], while the extracellular domain of NKG2D was used to target NKG2DL (Fig. 1B). Our BiTEs CAR-T possess advantages of low immunogenicity and high stability.

BiTEs CAR-T was generated as observed by MSLN CAR expression (Fig. 1C) and BiTEs secretion in culture supernatants using Western blot and ELISA (Fig. 1D, S2A). Transduction efficiency of BiTEs CAR-T was lower than MSLN CAR-T (Fig. S2B), CD4/CD8 ratios remain unchanged (Fig. S2C). Secreted BiTEs efficiently bind to CD3 on T cells and NKG2DL on tumor cells (Fig. 1E). To evaluate cytotoxicity, MICA-overexpressing MDA-MB-231 (MDA-MB-231^MICA^, Fig. S2D) cells were generated. BiTEs-containing supernatant enhanced untransduced T (UTD) cells cytotoxicity and activation, as indicated by increased CD69 expression and cytokine secretion (Fig. S2E). BiTEs CAR-T showed higher cytotoxicity against MDA-MB-231^MICA^ and higher CD69 expression than MSLN CAR-T (Fig. S3A, B). The secreted IFN-γ, TNF-α, granzyme A, granzyme B, perforin and granulysin were elevated (Fig. S3C). Further assessment using MICA-overexpressing 293T (293T^MICA^) and MSLN-overexpressing 4T1 (4T1^MSLN^) cells (Fig. S3D, E) demonstrated that BiTEs CAR-T was cytotoxic only against NKG2DL + cells (Fig. S3F, G). BiTEs CAR-T also showed higher cytotoxicity against MDA-MB-231^MICA^ compared to wild-type MDA-MB-231 (Fig. S3A, H).

We then evaluated BiTEs CAR-T activity in tumor cells with varying levels of MSLN and NKG2DL expression, including MDA-MB-468, HCT116, HeLa and MSLN-overexpressing MDA-MB-231 (MDA-MB-231^MSLN^) cells (Fig. S4A–C). BiTEs CAR-T exhibited higher cytotoxicity than MSLN CAR-T (Fig. S4D-G), and increased IFN-γ and TNF-α secretion (Fig. S4H, I). To simulate antigen heterogeneity, we mixed 20% MDA-MB-231^MSLN^ and 80% MDA-MB-231^MICA^ (MDA-MB-231^MICA/MSLN^). BiTEs CAR-T exhibited superior cytotoxicity across different E: T ratio (Fig. 1F) and induced higher levels of IFN-γ, TNF-α, IL-2, granzyme B and granulysin than MSLN CAR-T (Fig. 1G), confirmed by intracellular staining (Fig. 1H, I). CD25 and CD69 expression indicated enhanced T cell activation (Fig. 1J, K). Similar results were observed using 1:1 mixture of MDA-MB-231^MSLN^ and MDA-MB-231^MICA^ cells (Fig. S5).

**Fig. 1:**
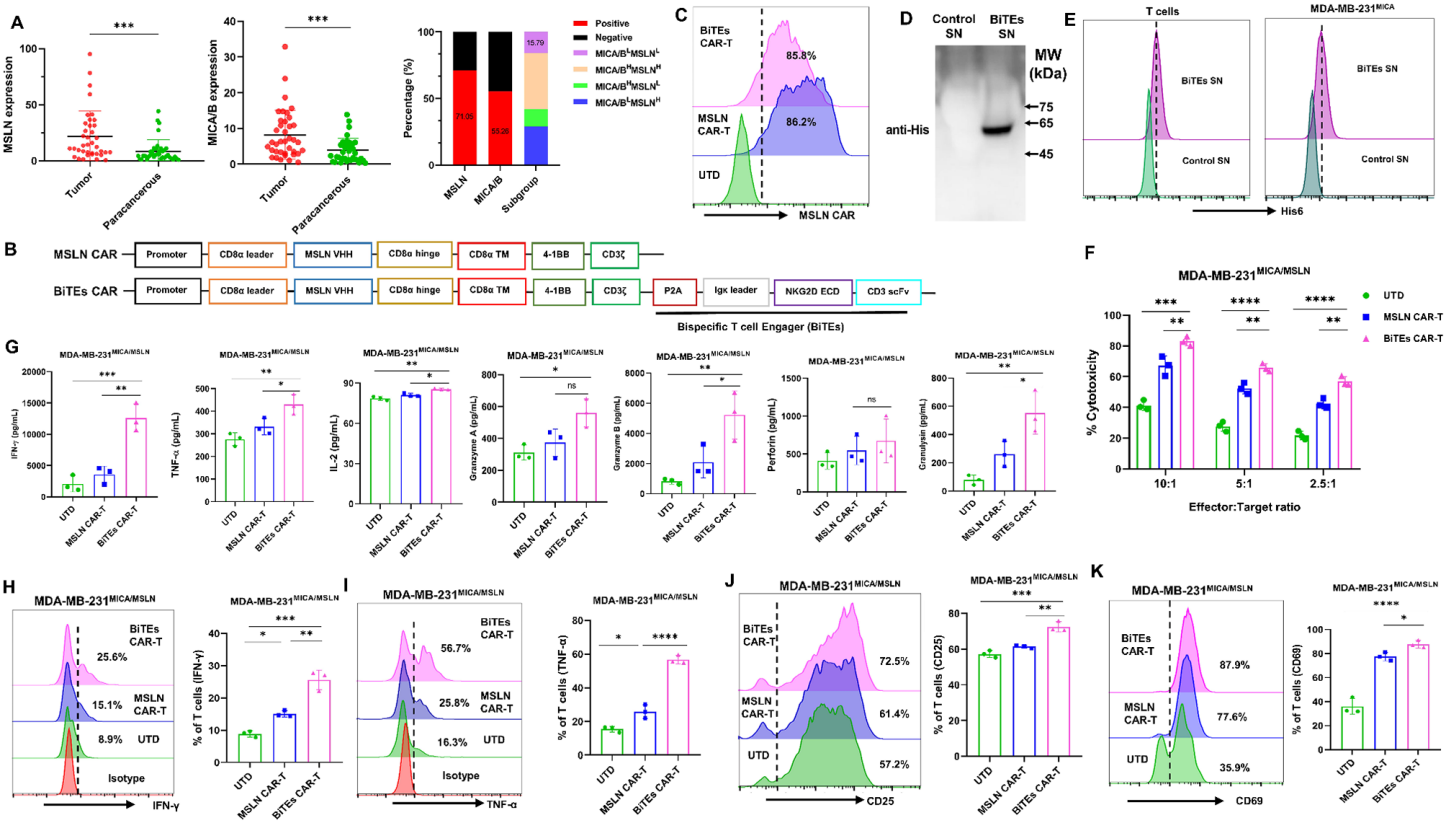
BiTEs CAR-T cells are efficacious against TNBC cells with heterogeneous antigens in vitro. **(A)** Expression (H-score) of Mesothelin (MSLN) and MICA/B in TNBC tissues and proportion of MSLN/MICA/B-positive patients in clinical cohort (*n* = 38). Subgroup analysis showed that the percentages of MICA/B^low^/MSLN^high^, MICA/B^high^MSLN^low^, MICA/B^high^/MSLN^high^, and MICA/B^low^/MSLN^low^ patients were 28.95%, 13.16%, 42.11%, and 15.79% respectively. **(B)** Schematic diagrams of MSLN CAR and BiTEs CAR constructs. **(C)** MSLN and BiTEs CAR expression of T cells on day 7 using FACS. **(D)** Western blot analysis for concentrated BiTEs from culture supernatants of T cells transduced with BiTEs CAR or control. **(E)** Flow cytometry analysis demonstrating his-tag detection of concentrated BiTEs binding to NKG2DL on MDA-MB-231^MICA^ and CD3 on T cells after incubation with BiTEs supernatant (SN). **(F)** The cytotoxic activity of UTD, MSLN CAR-T, and BiTEs CAR-T cells against MDA-MB-231^MICA/MSLN^ (a mixture of 80% MDA-MB-231^MICA^ and 20% MDA-MB-231^MSLN^) cells at E: T = 2.5, 5 and 10 using RTCA (*n* = 3). **(G)** MDA-MB-231^MICA/MSLN^ cells were cultured with UTD, MSLN CAR-T, or BiTEs CAR-T cells at an E: T ratio of 5:1 for 24 h. IFN-γ, TNF-α, IL-2, granzyme A, granzyme B, perforin and granulysin were measured using a LEGENDplex multi-analyte Flow Assay Kit (*n* = 3). **(H)** Intracellular flow cytometry analysis of IFN-γ secreting CD3^+^ T cells after co-culturing UTD, MSLN CAR-T, or BiTEs CAR-T cells and MDA-MB-231^MICA/MSLN^ cells at an E: T ratio of 5:1 for 24 h (*n* = 3). **(I)** Intracellular flow cytometry analysis of TNF-α secreting CD3^+^ T cells after co-culturing UTD, MSLN CAR-T, or BiTEs CAR-T cells and MDA-MB-231^MICA/MSLN^ cells at an E: T ratio of 5:1 for 24 h (*n* = 3). **(J)** Flow cytometry analysis of expression of CD25 on different CD3^+^ T cells after co-culture with MDA-MB-231^MICA/MSLN^ cells at an E: T ratio of 5:1 for 24 h (*n* = 3). **(K)** Flow cytometry analysis of expression of CD69 on different CD3^+^ T cells after co-culture with MDA-MB-231^MICA/MSLN^ cells at an E: T ratio of 5:1 for 24 h (*n* = 3). Each experiment was repeated at least twice with similar results. Representative data are shown. Statistical significance was considered as *P < 0.05, **P < 0.01, ***P < 0.001, and ****P < 0.0001, ns, not significant

A zebrafish xenograft MDA-MB-231^MICA/MSLN^ model was used to evaluate BiTEs CAR-T effect in vivo due to its efficiency and low cost [11]. BiTEs CAR-T significantly reduced tumor fluorescence intensity, confirming tumor regression (Fig. 2A–C). For the first time, we evaluated the efficacy of BiTEs CAR-T in a zebrafish model. We further assessed BiTEs CAR-T in an immunocompromised mouse model (Fig. 2D). BiTEs CAR-T effectively reduced tumor volume and prolonged survival compared to MSLN CAR-T (Fig. 2E, F), without significant weight loss (Fig. 2G). BiTEs CAR-T-treated mice showed higher levels of IFN-γ and increased CD3^+^ T cell percentages in blood samples (Fig. 2H, I, S6), indicating strong persistence and potency.

**Fig. 2:**
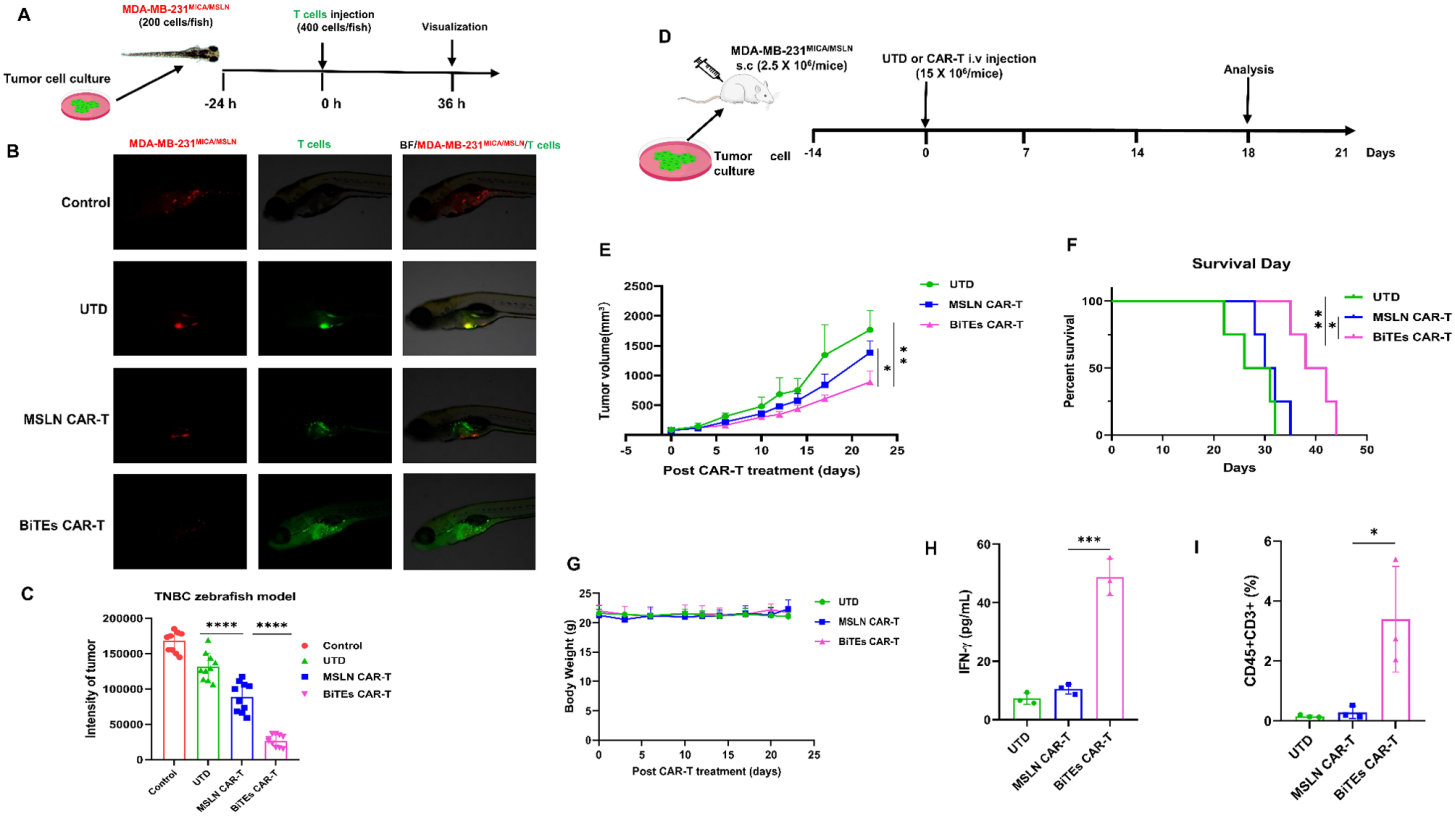
In vivo antitumor activity of BiTEs CAR-T cells against TNBC with heterogeneous antigens in zebrafish and mice models. **(A)** Schematic diagram of the zebrafish TNBC xenograft model. MDA-MB-231^MICA/MSLN^ (a mixture of 80% MDA-MB-231^MICA^ and 20% MDA-MB-231^MSLN^) cells were used to establish the TNBC model with heterogeneous antigens. **(B)** Cell fluorescence was visualized using fluorescent stereomicroscopy 36 h post T cells injection. **(C)** Statistical analysis of the fluorescence intensity of MDA-MB-231^MICA/MSLN^ tumor treated with UTD, MSLN CAR-T or BiTEs CAR-T cells in zebrafish model at 36 h post-treatment (*n* = 10). **(D)** Schematic diagram of the murine TNBC xenograft model. MDA-MB-231^MICA/MSLN^ cells were used to establish the TNBC model with heterogeneous antigens. **(E)** Growth curve of MDA-MB-231^MICA/MSLN^ xenograft treated with UTD, MSLN CAR-T or BiTEs CAR-T cells. **(F)** Kaplan-Meier survival curves of MDA-MB-231^MICA/MSLN^ tumor bearing-mice treated with UTD, MSLN CAR-T or BiTEs CAR-T cells (*n* = 4). **(G)** Body weight of MDA-MB-231^MICA/MSLN^ tumor bearing-mice treated with UTD, MSLN CAR-T or BiTEs CAR-T cells (*n* = 4). **(H)** The concentration of IFN-γ in blood samples of MDA-MB-231^MICA/MSLN^ tumor bearing-mice treated with UTD, MSLN CAR-T or BiTEs CAR-T cells (*n* = 3). **(I)** Percentage of transferred CD45^+^ CD3^+^ T cells in peripheral blood samples from tumor bearing-mice treated with UTD, MSLN CAR-T or BiTEs CAR-T cells (*n* = 3). Each experiment was repeated at least twice with similar results. Representative data are shown. Statistical significance was considered as *P < 0.05, **P < 0.01, ***P < 0.001, and ****P < 0.0001, ns, not significant

Despite promising results, BiTEs CAR-T efficacy in the mouse model was limited, likely due to tumor aggressiveness or CAR-T dosage [12]. Combining BiTEs CAR-T with chemotherapy or UTD may enhance the efficacy. Given the dual-targeting nature of BiTEs CAR-T, further toxicity assessments, including on-target off-tumor effects and cytokine release syndrome are essential. Overall, BiTEs CAR-T, with low immunogenicity and high stability, demonstrated superior antitumor activity against heterogeneous TNBC, highlighting its therapeutic potential.

## Electronic supplementary material

Below is the link to the electronic supplementary material.

Supplementary Material 1

## Data Availability

No datasets were generated or analysed during the current study.

## Abbreviations

BiTEs: Bi-specific T cells Engagers
CAR: Chimeric Antigen Receptor
MSLN: Mesothelin
NKG2DL: Natural Killer Group 2 member D Ligands
scFv: Single Chain Variable Fragments
TCGA: The Cancer Genome Atlas
TNBC: Triple Negative Breast Cancer
UTD: Untransduced T cells
VHH: Variable Heavy domain of Heavy Chain
