# Supplementary material for: Mesothelin-targeted CAR-T cells secreting NKG2D-BiTEs exhibit potent efficacy against triple-negative breast cancer
Source: Exp Hematol Oncol. 2025 Mar 3;14:27. doi: 10.1186/s40164-025-00621-y (PMC11874698; doi:10.1186/s40164-025-00621-y)
Supplement: Supplementary file 1 — Supplementary Material 1 [file 40164_2025_621_MOESM1_ESM.docx]

**Supplementary Material**

**Mesothelin-targeted CAR-T cells secreting NKG2D-BiTEs exhibit potent efficacy against triple-negative breast cancer**

Muhammad Auwal Saliu^a,b^, Qi Wang^a^, Mansur Dabai Salisu^a,b^, Yuanfeng Ren^c^, Pengchao Zhang^a,b^, Rabiatu Bako Suleiman^a,b^, Bingbing Cao^d^, Yiqiao Xu^d^, Xudong Liu^e^, Frederic Lluis^f^, Maoxuan Liu^a,b^*, Xiaochun Wan^a,b^*

^a^ Guangdong Immune Cell Therapy Engineering and Technology Research Center, Center for Protein and Cell-Based Drugs, Institute of Biomedicine and Biotechnology, Shenzhen Institutes of Advanced Technology, Chinese Academy of Sciences, Shenzhen 518055, China

^b^ University of Chinese Academy of Sciences, Beijing 100049, China

^c^ Department of Gastroenterology, Daping Hospital, Army Medical University, Chongqing, China.

^d^ Hunter Biotechnology, Inc. Hangzhou 310051, China

^e^ The State Key Laboratory for Complex, Severe and Rare Diseases, Peking Union Medical College Hospital, Beijing 100730, China

^f^ Department of Development and Regeneration, Stem Cell Institute, KU Leuven, Leuven 3000, Belgium

* Correspondence to:

Dr. Maoxuan Liu, [mx.liu@siat.ac.cn](mailto:mx.liu@siat.ac.cn); +86-755-86387944

Dr. Xiaochun Wan, [xc.wan@siat.ac.cn](mailto:xc.wan@siat.ac.cn); +86-755-86585218

**Materials and Methods**

**Cell lines and culture conditions**

The MDA-MB-231, MDA-MB-468, HCT116, HeLa and HEK293T cell lines were sourced from the American Type Culture Collection (ATCC, USA) and cultured in DMEM (Gibco, USA) media supplemented with 10% fetal bovine serum, FBS (Gibco, USA), 100 g/ml streptomycin, and 100 U/ml penicillin (Hyclone, USA). 4T1 cells were from the same source (ATCC) and cultured in RPMI-1640 (Gibco, USA) media supplemented with 10% FBS, streptomycin and penicillin. All the cells were incubated at 37 °C with 5% CO2.

**Immunohistochemistry (IHC) staining**

The TNBC tissue microarray comprising 38 TNBC tissues and paired adjacent noncancerous tissues was purchased from Wuhan Servicebio Technology Co., Ltd. (Wuhan, China). In brief, tissue slides were heated and deparaffinized in xylene immediately. After rehydration in a graded series of ethanol solutions, tissue slides were submerged in citrate antigen retrieval solution (pH=6.0) and heated in a microwave oven for antigen retrieval, and the activity of endogenous peroxidases was blocked by hydrogen peroxide. Subsequently, the sections were incubated with an anti-MICA/B mouse monoclonal antibody (Proteintech, 1:200 dilution) and anti-MSLN mouse monoclonal antibody (Invitrogen, 1:200 dilution) overnight at 4°C. After washing, the slides were incubated with HRP conjugated anti-mouse IgG (Servicebio, 1:200). Finally, the samples were counterstained with hematoxylin and dehydrated. The percentage of stained area was scored as follows: 0 (< 1%), 1 (1–25%), 2 (26–50%), 3 (51–75%), and 4 (75–100%). Staining intensity was scored as 0 (no staining), 1 (light brown), 2 (brown), and 3 (dark brown). Percentage and intensity scores were multiplied for each case. Negative (-) was assigned when the score was 0, “low expression” when scores were 1–3, “medium expression” when scores were 4–7, and “high expression” when scores were 8–12. In addition, Histochemistry score (H-score) was used to compare tumor and paracancerous tissues. H-Score（∑（pi×i）=（percentage of weak intensity cells ×1) + (percentage of moderate intensity cells ×2) + (percentage of strong intensity cells ×3), in which “i” indicates the classification of positive area: negative without coloration, 0 points; weak positive pale yellow, 1 point; moderate positive brownish-yellow, 2 points; strong positive tan counts 3 points. Pi represents the percentage of positive area for the corresponding grade [1].

**Plasmid construction and lentiviral production**

The VHH sequence targeting Mesothelin (MSLN) was derived from the Nb-C6 antibody (US Patent: US 20180002439A1) and was synthesized by Genewiz (China). VHH was fused to a CAR backbone comprising a human CD8a hinge spacer and transmembrane domain, 4-1BB costimulatory domain, and CD3ζ. The entire encoding sequence of the CAR expression molecule was cloned into a lentiviral vector pWPXLd (Addgene, #12258). BiTEs against NKG2DL and CD3 were designed and flanked by an Ig κ leader peptide and a His-tag element. The sequence of NKG2D extracellular domain was from aa 72–216 of human NKG2D (Uniprot P26718). The sequence of anti-CD3 scFv were obtained from sequence for publicly available blinatumomab. For the lentiviral production, the lentiviral plasmids were co-transfected into HEK293T cells using PEIpro (Polyplus, France) with the packaging plasmids psPAX2 and pMD2.G (Addgene) at a ratio of 5:3:2. Lentivirus was harvested 48h after transfection and filtered through a 0.45-µm filter. The lentiviral supernatants were used directly or stored at -80°C and thawed immediately before transduction.

**Generation of CAR-T cells**

The CAR-expressing lentiviral vectors were transduced into human primary T cells as described previously [2]. T cells were isolated from peripheral blood mononuclear cells (PBMCs) obtained from healthy donors and activated with Dynabeads™ CD3/CD28 (Thermo Fisher Scientific, USA) at 1:1 ratio and then cultured in Corning^®^ KBM 581 serum free medium containing IL-2 (50 U/mL, Novoprotein). After 24 h, activated T cells were transduced with the lentiviral particles. Seven days after stimulation, the beads were removed. CAR-T cells or UTD cells were permitted to expand until day 14 and subsequently transferred to storage in liquid nitrogen before functional assays. The percentage of CAR+ cells was used to calculate transduction efficiency using flow cytometry. All the studies involving human subjects were approved by the Institutional Review Board at Shenzhen Institutes of Advanced Technology, Chinese Academy of Sciences with written informed consent obtained from participants and conducted in accordance with the international ethical guidelines for biomedical research involving human subjects.

**Generation of target cells**

Lentiviral vectors encoding human MSLN or MICA were used separately to transduce MDA-MB-231 cells. Lentiviral supernatant containing MICA was also used to transduce 293T cells, while lentiviral supernatant containing MSLN was used to transduce 4T1 cells. After transduction, MDA-MB-231, 293T or 4T1 cells overexpressing either MSLN or MICA (MDA-MB-231^MICA or^ MDA-MB-231^MSLN^, 293T^MICA^, 4T1^MSLN^) were sorted using flow cytometry for further experiments.

**Flow cytometry analysis**

Cells were collected, washed twice with PBS, and suspended at a density of ~1×10^6^ cells/mL in cold PBS containing 0.5% BSA. Subsequently, labeled primary and secondary antibodies or direct antibodies were added to the cell suspension according to the manufacturer's instructions and incubated at 4 °C in the dark for 30 mins. We performed flow cytometry for T cells analysis using antibodies specific to human CD3 (BV421), CD4 (FITC), CD8 (APC), CD45 (PE), CD25 (PE), CD69 (APC), IFN-γ (PE), TNF-α (APC) (all from BioLegend, USA). Biotin labeled goat anti-Alpaca IgG antibody (Jackson, USA) and APC-streptavidin antibody (BioLegend, USA) were used as primary and secondary antibodies to detect CAR expression. Recombinant human NKG2D-Fc (Novoprotein, China) was used as primary antibody to detect NKG2DL expression and APC rat anti-human IgG Fc antibody (BioLegend, USA) served as the secondary antibody. Anti-human MSLN (BioLegend, USA) was used as primary antibody to detect MSLN expression and APC Goat anti-mouse IgG antibody (BioLegend, USA) served as the secondary antibody. Similarly, BiTEs supernatant or control supernatant concentrated by ultrafiltration columns were incubated with MDA-MB-231^MICA^ or T cells for about 1 h. The cells were stained with anti-His antibody (AlpVHHs, China) and detected with flow cytometer. All flow cytometry measurements were acquired with the CytoFLEX flow cytometer (Beckman, USA) and analyzed with FlowJo software.

**BiTEs quantification**

Protein concentrations of cell-free, BiTE-containing supernatant were determined using the His Tag ELISA Detection Kit (Jiangsu Meimian, China). Briefly, BiTEs CAR-T and UTD cells were cultured and allowed to grow for 14 days and supernatant was collected and analyzed intermittently.

**Western blotting**

T cells expressing BiTEs CAR were cultured for a week as well as untransduced T (UTD) cells to serve as control. The supernatant of the culture media was collected and concentrated using ultrafiltration columns with a molecular weight cutoff value of 10 kDa. The samples were treated with loading buffer and boiled at 100 ^o^C for 10 mins to denature the protein. The electrophoresis apparatus was assembled and run for conditions set at 60 V for 30 mins (upper gel) and 100V for 120 mins (lower gel). After the electrophoresis, the PVDF membrane was transferred and sealed using methanol, washing and sealing solution respectively and placed on a shaking device at room temperature for 60 mins. After the sealing is completed, horseradish peroxidase anti His-tag antibody was added and incubated overnight while shaking at 4 ^o^C, the membranes were then washed with TBST buffer three times, each time for 10 mins and developed in the darkroom.

**Cytotoxicity assay of CAR-T cells in vitro**

To assess the in vitro cytotoxicity of CAR-T cells against target cells, the xCELLigence RTCA SP instrument (ACEA Biosciences) was used as described previously [2]. Target cells were seeded at a density of 5×10^3^ cells/well into the plate and cultured for ~24 h. Data recording was paused and CAR-T normalized for transduction efficiency or UTD cells were added into the target cells at an indicated effector: target (E: T) ratio in triplicates. The cytotoxic activity based on the viability of attached target cells was monitored for at least 24 h, as reflected by cell index (CI) values. CI was normalized at the end of the experiment to remove any well-well variation using the RTCA software Pro (version 2.3.0). Percentage cytotoxicity was calculated at the end of the experiment as:

% Cytotoxicity = ((CI no effector - CI effector) / (CI no effector)) × 100.

**Cytokine measurements**

CAR-T or UTD cells were co-cultured with pre-seeded target cells at a 5:1 E:T ratio. After 24 h, supernatants were collected. Cytokine levels were measured using a 7-plex LEGENDplex multi-analyte Flow Assay Kit (BioLegend, USA). The experimental procedures were performed following the manufacturer's instructions. Analyses were performed using an LSRFortessa flow cytometer and data analysis software (BioLegend, USA).

**Zebrafish TNBC model in vivo**

Zebrafish of AB strain was sourced from Hunter Biotechnology, Inc. (Hangzhou, China). The xenograft transplantation model was established as described previously [3]. MDA-MB-231^MICA/MSLN^ (a mixture of 80% MDA-MB-231 overexpressing MICA and 20% MDA-MB-231 overexpressing MSLN) cells were stained with a red-fluorescent lipophilic membrane dye Dil (5 μM; 1,1’-dioctadecyl-3,3,3’,3’-tetramethylindocarbocyanine perchlorate), whereas CAR-T or UTD cells were stained with green-fluorescent lipophilic dye Dio (3,3’-dioctadecyloxacarbocyanine perchlorate). The labelled target cells were injected into the vitellicle of juvenile zebrafish 48 h post-fertilization at a density of 200 cells per fish (n = 10). After 24 h, effector cells from three groups (UTD, MSLN CAR-T, and BiTEs CAR-T) were injected into the vitellicle of zebrafish (400 cells/fish). Zebrafish were then visualized and captured under a fluorescent stereomicroscope (Nikon, Tokyo, Japan) after 36 h treatment with effector cells. The fluorescence intensity of each fish was determined and quantitated using ImageJ software.

**In vivo xenograft mouse model**

Mouse experiments were performed in accordance with relevant guidelines and regulations and were approved by the Institutional Animal Care and Use Committee at Shenzhen Institutes of Advanced Technology. Mice were maintained under specific-pathogen-free conditions with daily cycles of 12-h light–12-h darkness and health monitoring were carried out on a regular basis. Six-week-old female B-NDG mice used in this study were purchased from Biocytogen (Beijing, China). A total of 2.5 x 10^6^ MDA-MB-231^MICA/MSLN^ cells in 100 µL PBS containing 50% Matrigel (Corning, USA) were inoculated subcutaneously in right flanks. At 14 days after tumor cell injection, 15 × 10^6^ CAR-positive T cells were injected i.v. via the tail vein. Tumor growth was measured with a vernier caliper. A tumor size of 2000 mm^3^ was considered an end event.

To obtain immune cells from mouse blood, 100 µL of blood was taken from the lateral tail vein in heparinized capillary tubes at Day 18. Plasma was collected after centrifugation at 500 g for 5 mins. Red blood cells (RBCs) were then lyzed using RBC lysis buffer (Beyotime, China) according to the manufacturer’s instructions. The remaining cells were then washed twice with PBS and stained with anti-human CD3 and anti-human CD45 antibodies, followed by flow cytometry analysis. The interferon-gamma (IFN-γ) in plasma samples from mice was quantified by a LEGENDplex multi-analyte Flow Assay Kit (BioLegend, USA) according to the manufacturer’s instructions.

**Data analysis**

Experimental data obtained from this study were repeated at least twice or more independent experiments with at least triplicates for each analysis. Data were presented as mean ± SD and analyzed using GraphPad Prism version 8.0.1 statistical software. The comparison of variables between two groups was tested using student t-test while comparison between multiple (3 groups or more) was conducted using one-way ANOVA. Statistically significant difference was considered as *P < 0.05, **P < 0.01, ***P < 0.001, and ****P < 0.0001, ns, not significant.





**Fig. S1.** Analysis of expression of MSLN and NKG2DL in TNBC. **(A)** mRNA expression analysis of MSLN and NKG2DL in TNBC based on the TCGA datasets using GEPIA2. **(B)** Correlation analysis of MSLN and NKG2DL expression using GEPIA2. **(C)** Representative photomicrographs of MSLN and MICA/B expression in TNBC and normal tissues. The scale bar represents 100 µm in tumors and controls.

**

**

**Fig. S2.** BiTEs CAR-T phenotype and its function. BiTEs supernatant improved T cells cytotoxicity, activation and cytokine secretion after co-culture with MDA-MB-231 cells. **(A)** BiTEs concentration in supernatant increases over time. UTD T cells (green), or those transduced with BiTEs CAR (purple), were cultured with supernatant collected for His-tag ELISA analysis on days 7 and 11. **(B)** MSLN CAR and BiTEs constructs demonstrated efficient transduction of primary human T cells from five healthy donors. **(C)** Flow cytometry analysis of expression of CD8/CD4 on different CD3^+^T cells during culture. Graphs depicting cell surface markers used to phenotype CD8/CD4 CAR­T cell populations and CD4/CD8 ratio during culture. **(D)** Analysis of NKG2DL (MICA) overexpression on MDA-MB-231 cells by flow cytometry. **(E)** The cytotoxic activity of T cells cultured with BiTEs supernatant (SN) or control SN against MDA-MB-231^MICA^ cells at E: T = 9 and 18 using RTCA (n = 3). Flow cytometry analysis of CD69 expression on T cells cultured with BiTEs SN or control SN after co-culture with MDA-MB-231^MICA^ cells at an E:T ratio of 9:1 for 24 h (n = 3). MDA-MB-231^MICA^ cells were cultured with T cells and BiTEs SN or control SN at an E:T ratio of 9:1 for 24 h. IFN-γ and TNF-α were measured using a LEGENDplex multi-analyte Flow Assay Kit (n = 3). Each experiment was repeated at least twice with similar results. Representative data are shown. Statistical significance was considered as *P < 0.05, **P < 0.01, ***P < 0.001, and ****P < 0.0001, ns, not significant.

**

**

**Fig. S3.** BiTEs CAR-T cells are efficacious against NKG2DL-positive cells. **(A)** The cytotoxic activity of UTD, MSLN CAR-T and BiTEs CAR-T cells against MDA-MB-231^MICA^ cells at E:T = 2.5, 5 and 10 using RTCA (n = 3). **(B)** Flow cytometry analysis of expression of CD69 on different CD3^+^T cells after co-culture with MDA-MB-231^MICA^ cells at an E:T ratio of 5:1 for 24 h (n = 3). **(C)** MDA-MB-231^MICA^ cells were cultured with UTD, MSLN CAR-T or BiTEs CAR-T cells at an E:T ratio of 5:1 for 24 h. IFN-γ, TNF-α, IL-2, granzyme A, granzyme B, perforin and granulysin were measured using a LEGENDplex multi-analyte Flow Assay Kit (n = 3). **(D)** Analysis of NKG2DL (MICA) overexpression and MSLN expression on 293T cells by flow cytometry. **(E)** Analysis of MSLN overexpression and NKG2DL expression on 4T1 cells by flow cytometry. **(F, G, H)** The cytotoxic activity of UTD, MSLN CAR-T and BiTEs CAR-T cells against 293T^MICA^, 4T1^MSLN^ and MDA-MB-231 cells at E:T = 2.5, 5 and 10 (n = 3). Each experiment was repeated at least twice with similar results. Representative data are shown. Statistical significance was considered as *P < 0.05, **P < 0.01, ***P < 0.001, and ****P < 0.0001, ns, not significant.

**

**

**Fig. S4.** BiTEs CAR-T cells are efficacious against MSLN and NKG2DL-positive cells. A panel of tumor cells were stained with anti-human MSLN or recombinant NKG2D-Fc antibodies with matched isotype controls and analyzed by flow cytometry. **(A)** Analysis of MSLN expression on MDA-MB-468, HCT116 and HeLa cells by flow cytometry. **(B)** Analysis of NKG2DL expression on MDA-MB-468, HCT116 and HeLa cells by flow cytometry. **(C)** Analysis of MSLN overexpression on MDA-MB-231 cells by flow cytometry. **(D, E, F, G)** The cytotoxic activity of UTD, MSLN CAR-T and BiTEs CAR-T cells against MDA-MB-468, HCT116, HeLa and MDA-MB-231^MSLN^ cells at E:T = 2.5, 5 and 10 (n = 3). **(H)** Intracellular flow cytometry analysis of IFN-γ secreting CD3^+^ T cells after co-culturing UTD, MSLN CAR-T, or BiTEs CAR-T cells and MDA-MB-468 cells at an E:T ratio of 5:1 for 24 h (n = 3). **(I)** Intracellular flow cytometry analysis of TNF-α secreting CD3^+^ T cells after co-culturing UTD, MSLN CAR-T, or BiTEs CAR-T cells and MDA-MB-468 cells at an E:T ratio of 5:1 for 24 h (n = 3). Each experiment was repeated at least twice with similar results. Representative data are shown. Statistical significance was considered as *P < 0.05, **P < 0.01, ***P < 0.001, and ****P < 0.0001, ns, not significant.

**

**

**Fig. S5.** BiTEs CAR-T cells are efficacious against heterogeneous TNBC cells. The cytotoxic activity of UTD, MSLN CAR-T and BiTEs CAR-T cells against MDA-MB-231^MICA/MSLN (1:1)^ (a 1:1 mixture of MDA-MB-231^MSLN^ and MDA-MB-231^MICA^) cells at E: T = 2.5, 5 and 10 (n = 3). Each experiment was repeated at least twice with similar results. Representative data are shown. Statistical significance was considered as *P < 0.05, **P < 0.01, ***P < 0.001, and ****P < 0.0001, ns, not significant.

**

**

**Fig. S6.** Flow cytometry analysis of transferred CD45^+^ CD3^+^ T cells in peripheral blood samples from tumor bearing-mice treated with UTD, MSLN CAR-T or BiTEs CAR-T cells.

**References**

1. Cheng M, Xu J, Ding K, Zhang J, Lu W, Liu J, et al. Attenuation of relapsing fever neuroborreliosis in mice by IL-17A blockade. Proc Natl Acad Sci U S A. 2022;119(42):e2205460119.

2. Cao G, Zhang G, Liu M, Liu J, Wang Q, Zhu L, et al. GPC3-targeted CAR-T cells secreting B7H3-targeted BiTE exhibit potent cytotoxicity activity against hepatocellular carcinoma cell in the in vitro assay. Biochemistry and Biophysics Reports. 2022;31:101324.

3. Zhou Z, Li J, Hong J, Chen S, Chen M, Wang L, et al. Interleukin-15 and chemokine ligand 19 enhance cytotoxic effects of chimeric antigen receptor T cells using zebrafish xenograft model of gastric cancer. Front Immunol. 2022;13:1002361.
